# Leptin Modulates the Expression of miRNAs-Targeting POMC mRNA by the JAK2-STAT3 and PI3K-Akt Pathways

**DOI:** 10.3390/jcm8122213

**Published:** 2019-12-14

**Authors:** Adel Derghal, Julien Astier, Flavie Sicard, Charlène Couturier, Jean-François Landrier, Lourdes Mounien

**Affiliations:** 1 Aix Marseille Univ, INSERM, INRA, C2VN, 13005 Marseille, France; adel.der@gmail.com (A.D.); julien.astier@univ-amu.fr (J.A.); flavie.SICARD@univ-amu.fr (F.S.); charlene.COUTURIER@univ-amu.fr (C.C.); jean-francois.landrier@univ-amu.fr (J.-F.L.); 2 CriBioM, Criblage Biologique Marseille, Faculté de Médecine de la Timone, 13005 Marseille, France

**Keywords:** micro-RNA, POMC, leptin, stat3, AKT, ERK

## Abstract

The central control of energy balance involves a strongly regulated neuronal network within the hypothalamus and the brainstem. In these structures, pro-opiomelanocortin (POMC) neurons are known to decrease food intake and to increase energy expenditure. Thus, leptin, a peripheral signal that relays information regarding body fat content, modulates the activity of POMC neurons. MicroRNAs (miRNAs) are short non-coding RNAs of 22–26 nucleotides that post-transcriptionally interfere with target gene expression by binding to their mRNAs. It has been demonstrated that leptin is able to modulate the expression of miRNAs (miR-383, miR-384-3p, and miR-488) that potentially target POMC mRNA. However, no study has identified the transduction pathways involved in this effect of leptin on miRNA expression. In addition, miRNAs targeting POMC mRNAs are not clearly identified. In this work, using in vitro models, we have identified and confirmed that miR-383, miR-384-3p, and miR-488 physically binds to the 3′ untranslated (3′UTR) regions of POMC mRNA. Importantly, we show that leptin inhibits these miRNAs expression by different transduction pathways. Taken together, these results allowed us to highlight the miRNA involvement in the regulation of POMC expression downstream of the leptin signaling and satiety signal integration.

## 1. Introduction

The regulation of energy expenditure involved hormonal (leptin and insulin), metabolic (nutrients), and nervous signals [1,2]. The various signals mentioned before are integrated by the hypothalamus and the brainstem [1,2]. In this process, leptin has been shown to exert its satietogenic effect by stimulating the pro-opiomelanocortin (POMC) neurons located in the hypothalamic arcuate nucleus and nucleus of solitary tract in the brainstem [1,2,3,4]. Leptin is a polypeptide hormone (16 KDa) encoded by the Ob gene [5]. Leptin bind the long isoform of its receptor (LepRb) located on the membrane of POMC neurons [6,7]. After binding of leptin, three distinct cellular transduction pathways are stimulated by the Janus kinase (JAK2) protein [8,9]. Initially, JAK2 phosphorylates intracellular chain of the receptor on threonine amino-acid that are binding site for cytoplasmic proteins such as signal transducer and activators of transcription 3 (STAT3) [8,9]. STAT3 proteins form homodimers that are transported into the nucleus where they bind to specific DNA sequences, and activate the transcription of target genes such as that of POMC [8]. A large number of studies demonstrated that the JAK2 / STAT3 pathway is essential for the anti-obesity effects of leptin [1,8,9]. It has been also established that Mitogen activated kinase 1/2 (MEK 1/2)/ERK pathways plays an important role in the anti-obesity and cell differentiation effect of leptin [10,11]. Finally, the last activated pathway involves protein kinase B (Akt), which is common to the insulin pathway. Indeed, this pathway activates the Insulin receptor substrate (IRS) then a Phosphoinositide 3-kinase (PI3K) and is involved in metabolism, angiogenesis, and carcinogenesis [1,10]. In response to this activation, Akt will repress the transcription factor forkhead box 01 (FoxO1) which in turn inhibits the transcription of genes such as POMC [1,12]. It has recently been demonstrated that the activity of POMC neurons may involve post-transcriptional mechanisms such as the micro-ribonucleic acid (miRNAs) [13,14,15,16,17].

The miRNAs are important cellular regulators at the post-transcriptional level [18,19,20]. They represent a class of small, 22–26-nucleotide-long, noncoding RNA molecules that are involved in a large number of physiological functions such as energy metabolism, growth, cell differentiation, or apoptosis [18,19,21,22]. These noncoding RNAs behave as specific gene silencers by base pairing to 3ʹ untranslated region (3′UTR) of target messenger mRNAs but have been also proved to bind anywhere along the length of the mRNA transcript to exert their effects [23,24,25]. The miRNAs exert their action by inhibiting translation and by affecting mRNA stability and degradation [21,26]. The expression of miRNAs can be activated or repressed by transcription factors (TFs) [27]. However, few studies described how the miRNAs can be regulated by TFs. And particularly, the role of leptin in the modulation of miRNAs targeting POMC 3′UTR has never been assessed.

We have previously identified by in silico approach three miRNAs that could target the POMC and whose expression is under the control of leptin [13]. In particular, it has been shown that the expression of miR-383, miR-384-3P, and miR-488 that potentially target the 3′UTR portion of POMC is significantly increased in the hypothalamus from leptin-deficient *ob/ob* mice compared to wild-type mice [13]. In addition, the administration of leptin in *ob/ob* mice restored miRNA expression to levels similar to control animals [13]. All of these results suggest that leptin inhibits the expression of miR-383, miR-384-3P, and miR-488. Interestingly, Li et al. exhibited that multiple signaling pathways mediate the effect of leptin on miR-27a/b-3p [28]. All of these observations raise the original question of the signaling pathways associated with the inhibitory effect of leptin on the expression of our miRNAs of interest (i.e., miR-383, miR-384-3P, and miR-488). Then, we used the mHypoA-POMC/GFP cell line in order to clarify the implication of leptin-associated transduction pathways in the modulation of miRNAs-targeting POMC 3′UTR.

## 2. Experimental Section

### 2.1. Cell Culture and Reagents

The mHypoA-POMC/GFP cell line (CLU 500; Cedarlane, Burlington, ON, Canada) used in this study has been previously characterized [29]. Neurons were grown in DMEM (Dulbecco’s Modified Eagle’s Medium) (Gibco, Thermofisher Scientific, Illkrich, France), enriched with 5% fetal bovine serum (FBS, Hyclone, Fisher Scientific, Illkrich, France), 1% penicillin–streptomycin (Gibco, Thermofisher Scientific, Illkrich, France) and 4.5 g/L glucose. Neurons were cultured in 5% CO_2_ at 37 °C. HypoA-POMC/GFP neurons were grown in 6 well plates (TPP, Trasadingen, Switzerland) to ∼80–85% confluency. To examine the effect of leptin on the expression of POMC mRNA, treatment of mHypoA-POMC/GFP cell line with the recombinant leptin (Preprotech, Neuilly-sur-Seine, France) was performed. Final concentrations of leptin in culture medium were 1, 2.5, or 5 μM, and the cells were treated for 1.5 h or 3 h. The control group cells received vehicle alone. To identify signaling pathways involved in miRNAs regulation, mHypoA-POMC/GFP cells were treated or not for 24 h with specific inhibitors of STAT3 (cryptotanshinone (1 μM), Calbiochem, Merck-Millipore, Fontenay-sous-bois, France), AKT (MK 2206 (100 nM), Selleckchem, Euromedex, Souffelweyersheim, France), and ERK (PD 98059 (10 μM), Tocris, Rennes, France) pathways. Then the cells were treated with leptin ((5 μM), Preprotech, Neuilly-sur-Seine, France) for 2 h.

### 2.2. Quantitative RT-PCR (qRT-PCR) Analysis

Total RNA was extracted with TRI Reagent (Sigma-Aldrich, Saint-Quentin Fallavier, France). For RNA quantification, cDNA was synthetized by 2 μg total RNA with M-MLV Reverse Transcriptase (Promega, Charbonnières les bains, France). For miRNAs quantification, cDNA was synthetized by 1 μg total RNA by the qScript microRNA Quantification System (Quanta Biosciences, Beverly, MA, USA). For real-time PCR, we used LightCycler 480 (Roche, Indianapolis, IN, USA). We used Pomc forward primer 5′-CCCGCCCAAGGACAAGCGTT-3′ and reverse 5′-CTG GCCCTTCTTGTGCGCGT-3′; GAPDH (Glyceraldehyde 3-phosphate dehydrogenase) forward primer 5′- TTCTCAAGCTCATTTCCTGGTATG-3′ and reverse primer 5′-GGATAGGGCCTCTCTTGCTCA-3′. PCR was initiated by one cycle of 95 °C for 10 min, followed by 40 cycles of 10 s at 95 °C, 30 s at 60 °C, and 10 s at 72 °C, followed by a holding at 40 °C. For miRNAs, U6 and Sno202 were used as normalizers for miRNA quantification. For U6 we used forward primer 5′-ATTGGAACGATACAGAGAAGATT-3′ and reverse primer 5′-GGAAC GCTTCACGAATTTG-3′; Sno202 forward primer 5′-CTTTTGAACCCTTTTCCATCTG-3′; mir-383 forward primer 5′-CAGATCAGAAGGTGACTGTG-3′; mir-384-3p forward primer 5′-TG TAAACAATTCCTAGGCAATGA-3′; mir-488 forward primer 5′-CCCAGATAATAGCACTCTCAA-3′; for the reverse primers, we used the Universal Primer (Quanta Biosciences, Beverly, MA, USA). We performed quantitative PCR according to the manufacturer’s instructions (Quanta Biosciences, Beverly, MA, USA).

### 2.3. Luciferase Reporter Assays

POMC 3′UTR cloned in pmirGlo vector (50 ng, Creative Biogene, Shirley, NY, USA) was cotransfected with miScript mimic (200 nM Qiagen, Les Ulys, France) in Cos-1 cells using jetPEI (Polyplus transfection, Illkrich, France) and Hiperfect Reagent (Qiagen, Les Ulys, France), respectively, according to the manufacturer’s instruction. More precisely, miRNA mimics and no-target control are mixed with Optimem medium (Gibco, Thermofisher Scientific, Illkrich, France) and HiPerFect Transfection reagent (Qiagen, Les Ulys, France) with a mimic: reagent ratio of 1:4, and to obtain a final concentration in well of 200 nM. Plasmids (pmirGlo-3′UTR-POMC and pmirGlo) are mixed with 150 mM NaCl and JetPEI (Polyplus transfection, Illkrich, France), with a ratio of 0.5 µL of reagent for 50 ng. After 15 min of incubation at room temperature, 25 µL of each complex are spotted into corresponding wells. Then, 2.5 × 10^4^ Cos-1 cells in normal growth medium (DMEM, 10% fetal calf serum, 1% penicillin–streptomycin), freshly trypsinized, are seeding into each well, on top of the transfection complexes. Renilla and firefly luciferase activities were measured 48 h after cotransfection with Dual-Glo LuciferaseAssay System (Promega, Charbonnières Les bains, France) on a Perkin Elmer VictorX4 (Perkin Elmer, Waltham, MA, USA). Empty vector was used for background measurement. Firefly activity was under the control of POMC RNA 3′UTR, and Renilla luciferase activity was used for normalization.

### 2.4. Gene Ontology and KEGG Pathways Analysis

The gene ontology biological process (GO) terms and The Kyoto Encyclopedia of Genes and Genomes (KEGG) pathway terms enriched in predicted target genes were determined using mirPath v.3 from DIANA TOOLS bioinformatics resources [30].

### 2.5. Statistical Analysis

All data are expressed as mean ± SEM. Statistical analysis was performed by using GraphPad 6.0 software (San Diego, CA, USA). Significant differences between the control and treated groups were determined using ANOVA followed by a PLSD Fischer post-hoc test; *p* < 0.05 was considered statistically significant.

## 3. Results

### 3.1. Effect of Leptin on POMC Expression in mHypoA-POMC/GFP Cell Line

**Figure 1:**
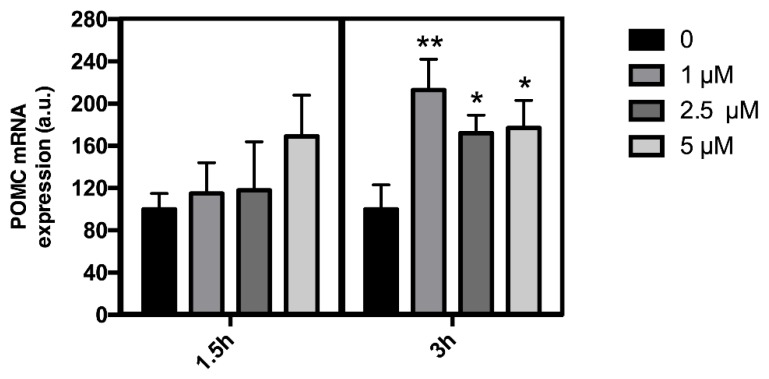
Effect of leptin on pro-opiomelanocortin (POMC) mRNA levels in the mHypoA-POMC/GFP cell line. Cells were incubated during 1.5 or 3 h in the absence (control; dark bars) or presence of different doses of leptin (1, 2.5, and 5 μM) (gray bars). The relative concentrations of POMC mRNA were determined by quantitative RT-PCR. GAPDH was used as the endogenous controls. The data are expressed as the mean ± SEM of at least three independent experiments. The values are presented as means ± SEM. * *p* < 0.05; ** *p* < 0.01.

qRT-PCR was performed in order to determine the effect of leptin on POMC mRNA levels in the mHypoA-POMC/GFP cell line. Treatment with graded concentrations of leptin induced a dose-dependent increase in POMC mRNA levels with a maximum effect observed at 1 μM leptin (+113%; *p* < 0.01) (Figure 1). Time-course experiments revealed that the effect of leptin on POMC mRNA levels was significant only for 3 h of treatment (Figure 1).

### 3.2. miR-383, miR-384-3p, and miR-488 Target POMC 3′UTR

**Figure 2:**
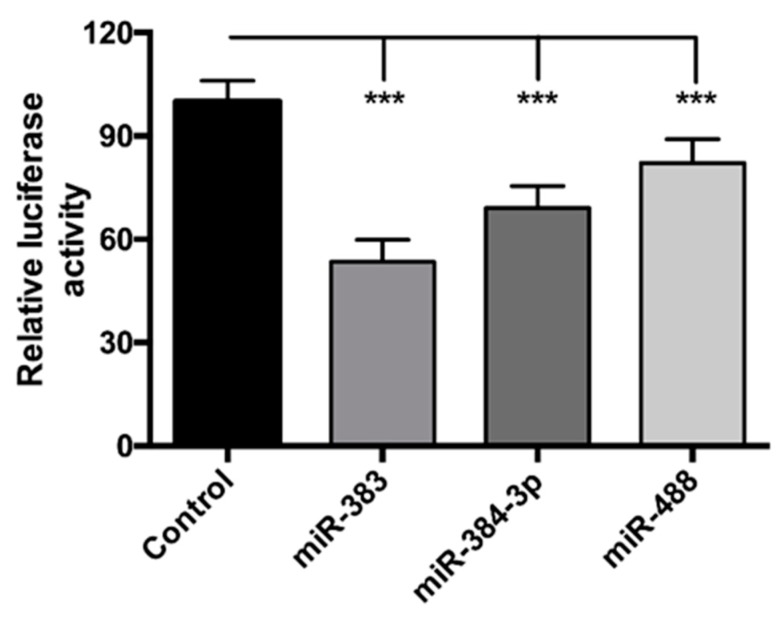
miR-383, miR-384-3p, and miR-488 directly target POMC. POMC 3′UTR cloned in pmirGlo vector was transfected with miR-383, miR-384-3p, or miR-488 mimic in Cos-1 cells for 48 h. miR-488 was used as a positive control for experiments. Renilla activity was under the control of POMC 3′UTR, and firefly activity was used for normalization. The data are expressed as the mean ± SEM of at least three independent experiments. The values are presented as means ± SEM. *** *p* < 0.001.

We have previously identified through in silico approach three miRNAs that could target the POMC and whose expression is under the control of leptin [13]. To confirm the effect of miR-383, miR-384-3p, and miR-488 on POMC, we transfected POMC 3′UTR with miR mimics. miR-488 was used as a positive control for the experiment, given that it has already been reported to target POMC [31,32]. The ratio of Renilla to firefly luciferase activity was significantly decreased for miR-383, miR-384-3p, and miR-488 (−47%, −31%, and −18%, respectively) (Figure 2), validating our experimental conditions, and strongly suggesting that POMC may be considered as a target of miR-383, miR-384-3p, and miR-488.

### 3.3. Regulation of the Expression of the miRNAs of Interest by Leptin via the STAT3, AKT, and ERK Pathways

**Figure 3:**
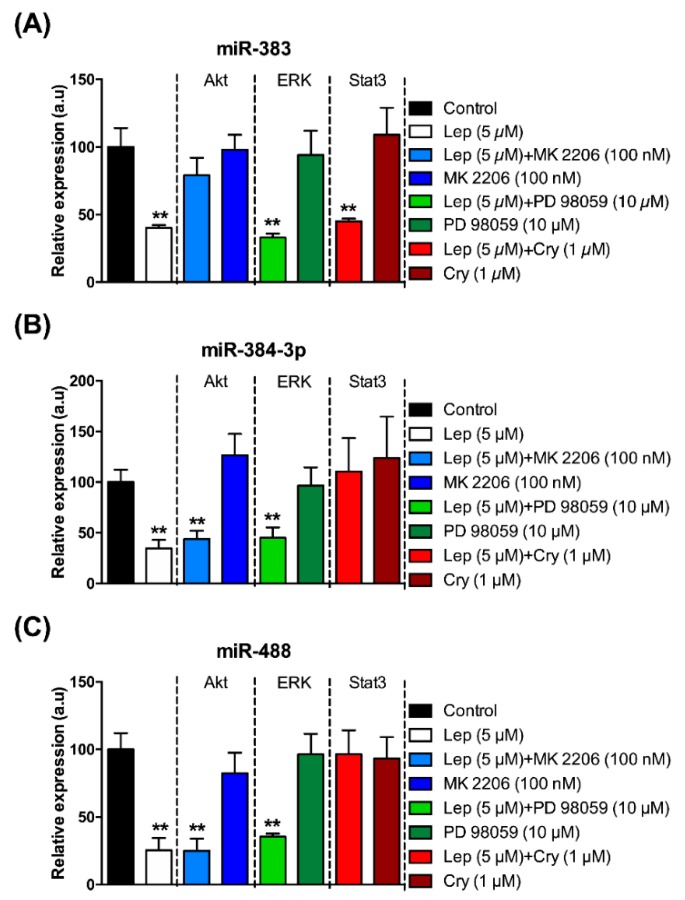
Leptin modulates the expression of the miRNAs of interest by different pathways. Effect of leptin on miR-383 (**A**), miR-384-3p (**B**), and miR-488 (**C**) expression after 24 h of pharmacological inhibition of JAK2-STAT3, PI3K-Akt, and MEK 1/2/ERK pathways. The data are expressed as the mean ± SEM of at least three independent experiments. The values are presented as means ± SEM. ** *p* < 0.01.

In order to determine if leptin regulates miRNAs expression in this model, mHypoA-POMC/GFP were treated with 5 μM leptin. This concentration was determined to be sufficient for the regulation of Pomc mRNA expression (Figure 1). Leptin treatment in mHypoA-POMC/GFP culture for 2 h significantly decreased miR-383, miR-384-3p, and miR488 levels (Figure 3A–C). Leptin is known to act primarily through the JAK2-STAT3, PI3K-Akt, and MEK 1/2/ERK pathways within POMC neurons [10]. Then to clarify the pathways involved in the regulation of miRNAs expression by leptin, cryptotanshinone (1 μM), MK 2206 (100 nM), and PD 98059 (10 μM) were used as specific blocker of JAK2-STAT3, PI3K-Akt, and MEK 1/2/ERK pathways, respectively. These concentrations were determined to be sufficient in regulating miRNAs expression without inducing significant cellular death. In the mHypoA-POMC/GFP cell line, pre-treatment with MK 2206 (100 nM) blocked the decrease of miR-383 in response to leptin (Figure 3A). The inhibitory effect of leptin on the expression of miR-383 was not blocked either by cryptotanshinone (1 μM) or PD 98059 (10 μM) (Figure 3A). For miR-384-3p and miR-488, inhibition of JAK2-STAT3 pathway by pre-treatment with cryptotanshinone (1 μM) for 24 h attenuated the decrease of miRNAs expression in response to leptin (Figure 3B,C). Pre-treatment with MK 2206 (100 nM) or PD 98059 (10 μM) has no effect on the inhibitory action of leptin on the expression of miR-384-3p and miR-488 (Figure 3B,C). Cryptotanshinone (1 μM), MK 2206 (100 nM), and PD 98059 (10 μM) had no effect alone on the expression of the miRNAs of interest (Figure 3A–C). These results suggest that leptin may influence miR-383, miR-384-3p, and miR-488 expression though different mechanism of action.

### 3.4. Pathway Analysis of miRNAs Targeting POMC

**Figure 4:**
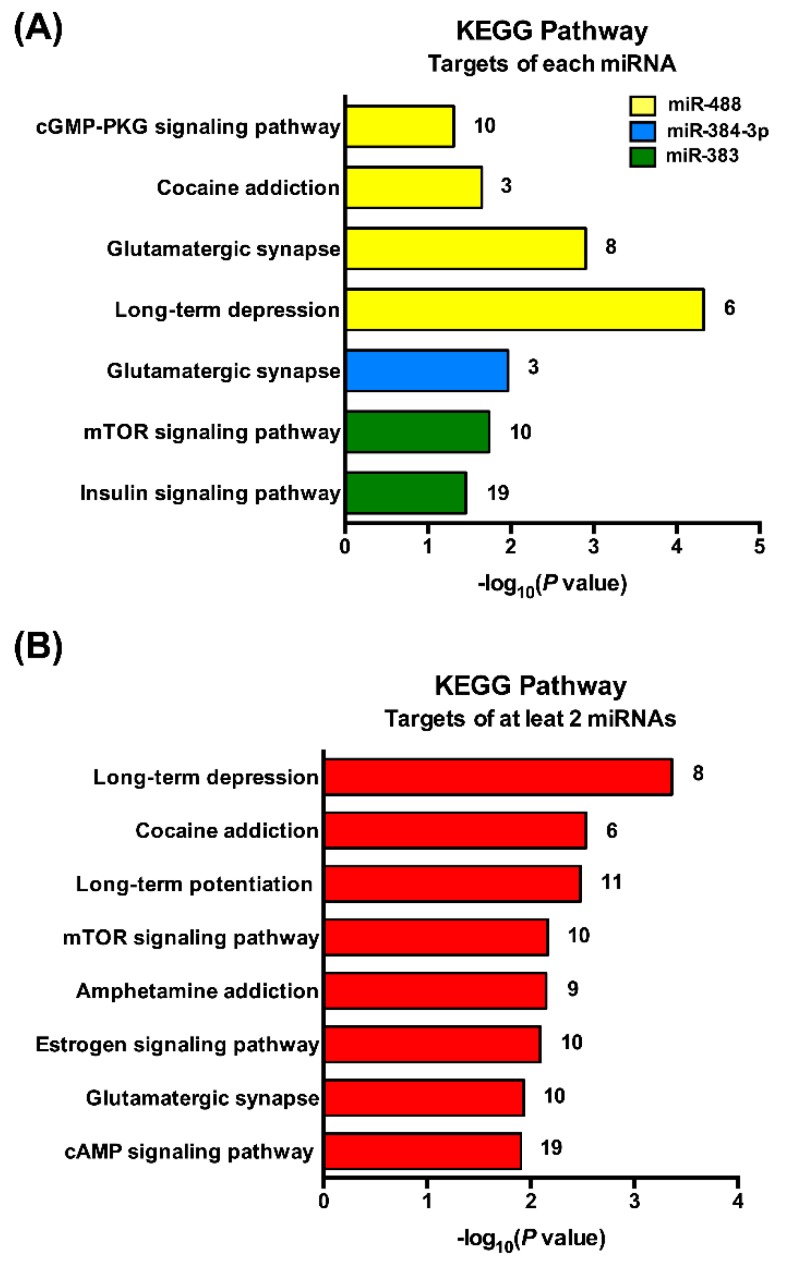
Kyoto Encyclopedia of Genes and Genomes (KEGG) pathway analysis associated with predicted target genes of the 3 miRNAs of interest. The Kyoto Encyclopedia of Genes and Genomes (KEGG) (http://diana.imis.athena-innovation.gr/DianaTools/index.php) was used to analyze the signaling pathways of specified miRNA target genes. (**A**) Bar plot ranking of the enrichment score (−log_10_ (*p* value)) values for significant enrichment pathways of each miRNA of interest. (**B**) Bar plot ranking of the enrichment score (−log_10_ (*p* value)) values for significant enrichment pathways of at least 2 miRNAs of interest. The number of target genes is indicated next to each bar.

To further study the functions and underlying mechanisms of these three miRNAs of interest, KEGG (http://diana.imis.athena-innovation.gr/DianaTools/index.php) was used to examine the signaling pathways of specified miRNA target genes. We found that the 3 miRNAs are involved in several pathways (Figure 4A). According to enrichment score, long-term depression (LTD), glutamatergic transmission, cocaine addiction and cGMP-PKG signaling pathway are the main signaling pathways associated with miR-488 (Figure 4A). The pathways most correlated with miR-384-3p and miR-383 were glutamatergic synapse, mTOR, and insulin signaling pathways (Figure 4A). In addition, we also examined the signaling pathways associated with simultaneously at least 2 miRNAs. This analysis revealed that the target genes of at least 2 miRNAs are associated with neurotransmission, addiction, and different signaling pathways (Figure 4B).

### 3.5. Gene Ontology Analysis of miRNAs Targeting POMC

**Figure 5:**
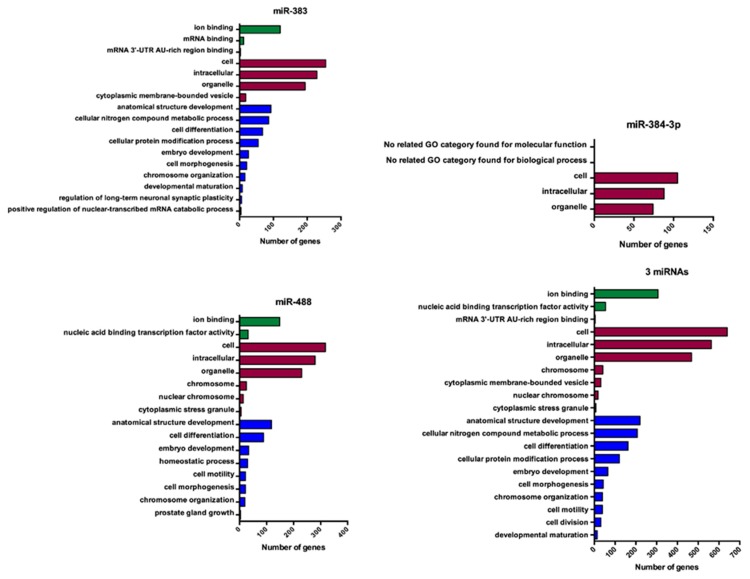
Gene ontology (GO) enrichment analysis of the 3 miRNAs of interest. The three GO classifications—molecular function (green bar), biological process (purple bar), and cellular component (blue bar)—were evaluated separately. The figure depicts the major biological pathways associated with the miRNAs of interest (alone or combined). GO-Analysis was performed based on mirPath v.3 from DIANA TOOLS bioinformatics resources.

Using GO-analysis, the functional categories associated with the miRNAs of interest were identified. We explored three domains: biological process (BP), cellular component (CC), and molecular function (MF). This analysis revealed that the genes targeted by miR-383 and miR-488 are involved in several important biological pathways (Figure 5). In contrast, genes targeted by miR-384-3p are involved only in CC domain (Figure 5). Finely, the genes targeted by the 3 miRNAs are involved in a large panel of biological functions (Figure 5).

## 4. Discussion

POMC neurons are essential for the integration of peripheral (nutrients and hormones) and central (neuropeptides and neurotransmitters) signals [1,2,33,34,35]. Recently, pharmacogenetic and optogenetic techniques have further proved that POMC neuronal activation reduces food intake and increases energy expenditure [36,37]. The important aim of current research is to clarify the molecular mechanism involved in the integration by POMC neurons of these multiple peripheral metabolic signals such as leptin. In this study, we also confirmed for that leptin modulates the expression of POMC in the mHypoA-POMC/GFP cell line which can be thus a good model for the mechanistic studies. This model has been validated in other studies [29,38,39,40].

It is essential to identify the intracellular mediators that allow these POMC neurons to respond to energy status modifications. In this context, we and others observed that miRNAs can be important intracellular mediators in the modulation of POMC neurons activity [1,13,14,15,16,17]. So far, only two teams have identified mir-488 as miRNA that physically interact with the 3′UTR of POMC mRNA [29,32]. For instance, they revealed that miR-488 can modulate the expression of POMC mRNA for animal coat control [32]. In our study, we demonstrated that mir-383 and miR-384-3p bind physically with 3′UTR of POMC mRNA. Many studies have depicted an important role of mir-383 in cancer and cell viability and proliferation [41,42,43]. Interestingly, Wang et al. suggested that miR-383 can protect against cognitive impairment and hippocampal neurons apoptosis induced by propofol [44]. In accordance with these studies, KEEG and GO analyses revealed that miR-383 targeting genes are important for important cell process. It also been suggested that miR-383 suppressed the PI3K-AKT-mTOR signaling pathway to inhibit development of cervical cancer via down-regulating Poly (ADP-ribose) polymerase-2 [45]. The KEEG analysis confirm that miR-383 target several genes involved in mTOR signaling pathways. In the context of feeding behavior, it has been clearly demonstrated that mTOR signaling pathway is essential for POMC neuron activity [46,47]. To date, few studies clarified the function of miR-384-3p. For instance, Xia et al. showed that miR-384-3p inhibited retinal neovascularization during diabetic retinopathy [48]. KEEG analysis also revealed that the miRNAs of interest control activity-dependent synaptic plasticity, such as long-term potentiation (LTP) and LTD. Interestingly, several studies highlighted that energy state may control the LTP as well as LTD [49,50]. Particularly, a tetanic stimulation protocol induced LTD at POMC neurons in fed mice but weak LTP in food-deprived mice [50]. Then, it is conceivable that miRNAs may be involved in the control of LTP and LTD in POMC neurons. Additional experiments could clarify this last point. Subsequently, a GO analysis was added to our study. The GO analysis included biological process, molecular function and cellular components. GO term analysis revealed that miR-383 and miR-488 were involved in a large number of process and functions as cell cycle or morphogenesis as well as organelle function. In accordance with this observation, the importance of miRNAs in the development of POMC neurons has been highlighted by the specific deletion of Dicer in POMC-expressing cells which led to a postnatal ablation of POMC neurons [15,16]. The GO analysis also revealed that miR-384-3p is involved in only few cellular components functions suggesting a minor role for this miRNA.

In a previous study, we have described that leptin modulated the expression of miR-383, miR-384-3p, and miR-488 [13]. Since the miRNAs can be activated or repressed by TFs, we explored the pathways involved in the inhibitory effect of leptin. The study of miRNA regulation by TFs has been relatively limited [27,51]. However, TFs such as p53, MYC, ZEB1 and ZEB2, and myoblast determination protein 1 (MYOD1) positively or negatively regulate miRNA expression [51,52,53]. By pharmacological approach, we showed that leptin modulates by different pathways the expression of miR-383, miR384-3p, and miR-488. The JAK2-STAT3 pathway is involved in the regulation of miR-384-3p and miR-488 by leptin while this signal modulates miR-383 via PI3K-Akt pathway. Interestingly, it has been shown that STAT3 down-regulated the expression of miR-383 in a skin cancer cell line [54]. This different observation can be explained by the fact that the cell line used in this study is different. Our pioneering study highlights the fact that leptin via the JAK2-STAT3 and PI3K-Akt pathways might modulate the expression of miRNAs targeting POMC. However, we need to deepen these observations. In particular, locations of the miRNA promoters have not yet been mapped for miR-383, miR-384-3p, and miR-488 genes but can be inferred from collective analysis of CpG islands and chromatin immunoprecipitation followed by sequencing (ChIP–seq) data [55].

## 5. Conclusions

Taken together, these results allowed us to highlight the miRNA involvement in the regulation of POMC expression downstream of the leptin signaling and satiety signal integration.
